# (*E*)-2-Bromo­methyl-3-(*o*-tol­yl)acrylo­nitrile

**DOI:** 10.1107/S1600536813021041

**Published:** 2013-08-03

**Authors:** J. Kanchanadevi, G. Anbalagan, R. Selvakumar, M. Bakthadoss, B. Gunasekaran, V. Manivannan

**Affiliations:** a Department of Physics, Velammal Institute of Technology, Panchetty, Chennai 601 204, India; b Department of Physics, Presidency College (Autonomous), Chennai 600 005, India; c Department of Organic Chemistry, University of Madras, Guindy campus, Chennai 600 025, India; d Department of Physics & Nano Technology, SRM University, SRM Nagar, Kattankulathur, Kancheepuram Dist, Chennai 603 203 Tamil Nadu, India; e Department of Research and Development, PRIST University, Vallam, Thanjavur 613 403, Tamil Nadu, India

## Abstract

The title compound C_11_H_10_BrN, has an *E* conformation at the C=C bond of the acrylo­nitrile unit. The vinyl group makes a dihedral angle of 44.53 (12)° with the benzene ring. In the crystal, weak C—H⋯π inter­actions involving the benzene ring are observed.

## Related literature

For the biological activity of cyano­acrylates, see: Zhang *et al.* (2009 ▶); Obniska *et al.* (2005 ▶); For related structures, see: Ye *et al.* (2009 ▶); Suresh *et al.* (2012 ▶).
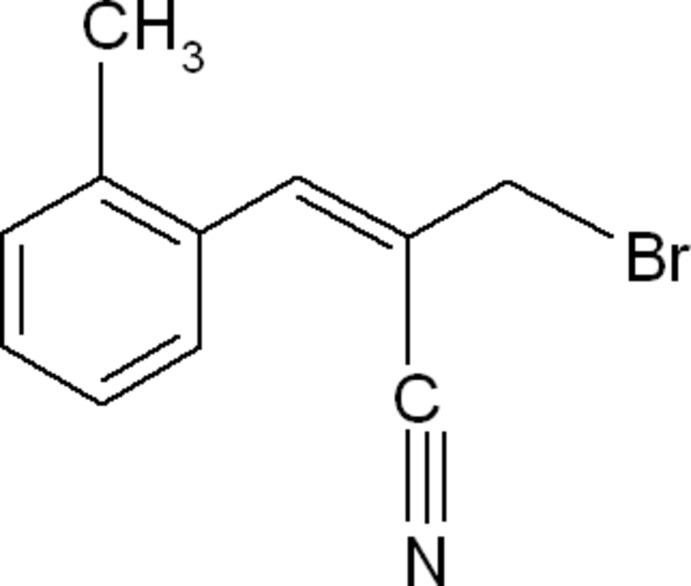


## Experimental

### 

#### Crystal data

C_11_H_10_BrN
*M* *_r_* = 236.11
Monoclinic, 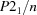

*a* = 7.5473 (8) Å
*b* = 11.7362 (10) Å
*c* = 11.5228 (11) Å
β = 96.436 (3)°
*V* = 1014.22 (17) Å^3^
*Z* = 4
Mo *K*α radiation
μ = 4.00 mm^−1^
*T* = 295 K
0.20 × 0.20 × 0.15 mm

#### Data collection

Bruker APEXII CCD diffractometer
Absorption correction: multi-scan (*SADABS*; Sheldrick, 1996 ▶) *T*_min_ = 0.435, *T*_max_ = 0.535
8718 measured reflections
1960 independent reflections
1261 reflections with *I* > 2σ(*I*)
*R*_int_ = 0.034

#### Refinement

*R*[*F* ^2^ > 2σ(*F* ^2^)] = 0.035
*wR*(*F* ^2^) = 0.085
*S* = 1.00
1960 reflections
119 parameters
H-atom parameters constrained
Δρ_max_ = 0.46 e Å^−3^
Δρ_min_ = −0.26 e Å^−3^

### 

Data collection: *APEX2* (Bruker, 2008 ▶); cell refinement: *SAINT* (Bruker, 2008 ▶); data reduction: *SAINT*; program(s) used to solve structure: *SHELXS97* (Sheldrick, 2008 ▶); program(s) used to refine structure: *SHELXL97* (Sheldrick, 2008 ▶); molecular graphics: *PLATON* (Spek, 2009 ▶); software used to prepare material for publication: *SHELXL97*.

## Supplementary Material

Crystal structure: contains datablock(s) I. DOI: 10.1107/S1600536813021041/gk2587sup1.cif

Structure factors: contains datablock(s) I. DOI: 10.1107/S1600536813021041/gk2587Isup2.hkl

Supplementary material file. DOI: 10.1107/S1600536813021041/gk2587Isup3.cml

Additional supplementary materials:  crystallographic information; 3D view; checkCIF report

## Figures and Tables

**Table 1:** Hydrogen-bond geometry (Å, °) *Cg*1 is the centroid of the C1–C6 ring

*D*—H⋯*A*	*D*—H	H⋯*A*	*D*⋯*A*	*D*—H⋯*A*
C7—H7⋯*Cg*1^i^	0.93	2.97	3.654 (7)	131
C11—H11*B*⋯*Cg*1^ii^	0.96	2.86	3.699 (1)	146

Symmetry codes: (i) 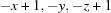; (ii) 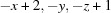.

**Fig. 1.**
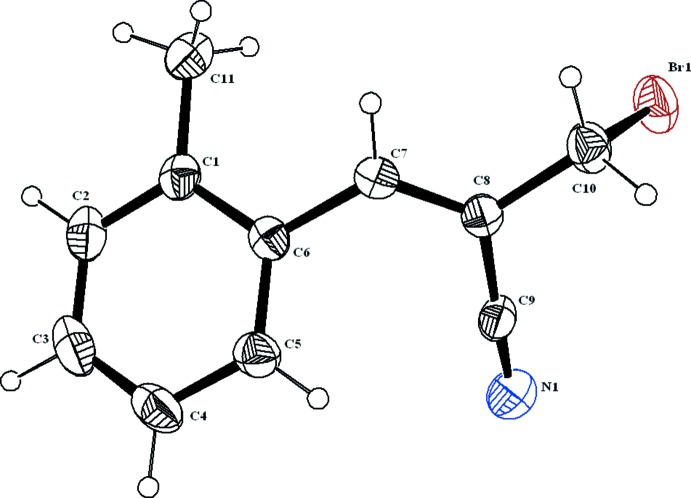
The molecular structure of (I), with atom labels and 30% probability displacement ellipsoids for non-H atoms.

**Fig. 2.**
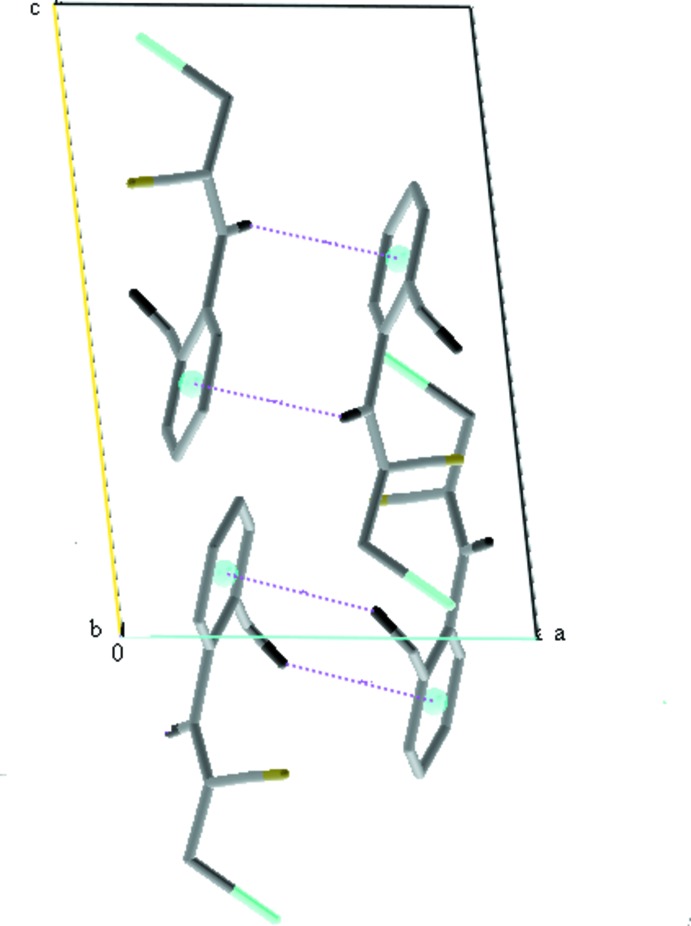
The packing diagram showing C-H···π interactions as dashed lines.

## supplementary crystallographic information

### 1. Comment

Cyanoacrylates and its derivatives have been widely used as agrochemicals (Zhang *et al.*, 2009) and are an important intermediate in drug synthesis (Obniska *et al.*, 2005).

The geometric parameters of the title molecule (Fig. 1) agree well with reported similar structure (Ye *et al.*, 2009; Suresh *et al.*, 2012). The vinyl group makes a dihedral angle of 44.53 (12) ° with the benzene ring. The acrylonitrile (C7–C8) and cyano (C9–N1) groups deviate from the mean plane of the benzene (C1–C6) ring.

The crystal packing is controlled by weak C—H···π [C7—H7···*Cg*1(1 - *x*, -*y*, 1 - *z*) distance of 3.654 (7) Å, C11—H11B···*Cg*1(2 - *x*, -*y*, 1 - *z*) distance of 3.699 (1) Å, (*Cg*1 is the centroid of the ring defined by the atoms C1—C6)] interactions.

### 2. Experimental

To a stirred solution of 2-[hydroxy(*o*-tolyl)methyl]acrylonitrile (1 equivalent) in dichloromethane (DCM) was added a 48% hydrobromic acid (2 equivalent) solution and then a concentrated sulfuric acid solution (catalytic amount) at 273 K. After stirring overnight at room temperature, the mixture was diluted with DCM and water. The aqueous phase was extracted twice with DCM. The combined organic phase was washed twice with water an then dried with sodiumsulfate. Removal of the solvent led to the crude product which was purified through a pad of silica gel (100–200 mesh) using ethylacetate and hexanes (1:9) as solvents. The pure title compound was obtained as a colorless solid (yield 90%; m.p. 383-387 K).

### 3. Refinement

H atoms were positioned geometrically and refined using riding model with C—H = 0.93 Å and *U*_iso_(H) = 1.2U_eq_(C) for aromatic C—H, C—H = 0.97 Å and *U*_iso_(H) = 1.2U_eq_(C) for CH_2_ and C—H = 0.96 Å and *U*_iso_(H) = 1.5U_eq_(C) for CH_3_.

### Crystal data

**Table:**

C_11_H_10_BrN	*F*(000) = 472
*M**_r_* = 236.11	*D*_x_ = 1.546 Mg m^−^^3^
Monoclinic, *P*2_1_/*n*	Mo *K*α radiation, λ = 0.71073 Å
Hall symbol: -P 2yn	Cell parameters from 1980 reflections
*a* = 7.5473 (8) Å	θ = 2.5–25.8°
*b* = 11.7362 (10) Å	µ = 4.00 mm^−^^1^
*c* = 11.5228 (11) Å	*T* = 295 K
β = 96.436 (3)°	Block, colourless
*V* = 1014.22 (17) Å^3^	0.20 × 0.20 × 0.15 mm
*Z* = 4	

### Data collection

**Table:**

Bruker APEXII CCD diffractometer	1960 independent reflections
Radiation source: fine-focus sealed tube	1261 reflections with *I* > 2σ(*I*)
Graphite monochromator	*R*_int_ = 0.034
Detector resolution: 0 pixels mm^-1^	θ_max_ = 25.9°, θ_min_ = 2.5°
ω and φ scans	*h* = −9→9
Absorption correction: multi-scan (*SADABS*; Sheldrick, 1996)	*k* = −14→13
*T*_min_ = 0.435, *T*_max_ = 0.535	*l* = −14→14
8718 measured reflections	

### Refinement

**Table:**

Refinement on *F*^2^	Primary atom site location: structure-invariant direct methods
Least-squares matrix: full	Secondary atom site location: difference Fourier map
*R*[*F*^2^ > 2σ(*F*^2^)] = 0.035	Hydrogen site location: inferred from neighbouring sites
*wR*(*F*^2^) = 0.085	H-atom parameters constrained
*S* = 1.00	*w* = 1/[σ^2^(*F*_o_^2^) + (0.036*P*)^2^ + 0.5483*P*] where *P* = (*F*_o_^2^ + 2*F*_c_^2^)/3
1960 reflections	(Δ/σ)_max_ < 0.001
119 parameters	Δρ_max_ = 0.46 e Å^−^^3^
0 restraints	Δρ_min_ = −0.26 e Å^−^^3^

### Fractional atomic coordinates and isotropic or equivalent isotropic displacement parameters (Å^2^)

**Table:**

	*x*	*y*	*z*	*U*_iso_*/*U*_eq_	
C1	0.7824 (4)	−0.0112 (3)	0.5604 (3)	0.0451 (8)	
C2	0.8374 (5)	0.0182 (4)	0.6742 (3)	0.0587 (10)	
H2	0.8886	−0.0372	0.7252	0.070*	
C3	0.8186 (5)	0.1269 (4)	0.7146 (3)	0.0702 (12)	
H3	0.8563	0.1443	0.7922	0.084*	
C4	0.7448 (5)	0.2096 (4)	0.6414 (3)	0.0634 (11)	
H4	0.7307	0.2831	0.6691	0.076*	
C5	0.6911 (5)	0.1840 (3)	0.5260 (3)	0.0508 (9)	
H5	0.6417	0.2406	0.4759	0.061*	
C6	0.7101 (4)	0.0752 (3)	0.4843 (3)	0.0398 (8)	
C7	0.6525 (4)	0.0464 (3)	0.3617 (3)	0.0411 (7)	
H7	0.5936	−0.0227	0.3480	0.049*	
C8	0.6760 (4)	0.1091 (3)	0.2682 (2)	0.0410 (8)	
C9	0.7740 (5)	0.2128 (3)	0.2782 (3)	0.0496 (9)	
C10	0.6063 (5)	0.0732 (3)	0.1480 (3)	0.0530 (9)	
H10A	0.5312	0.1331	0.1112	0.064*	
H10B	0.5336	0.0055	0.1521	0.064*	
C11	0.8037 (5)	−0.1309 (3)	0.5196 (3)	0.0582 (9)	
H11A	0.8550	−0.1767	0.5838	0.087*	
H11B	0.8807	−0.1316	0.4587	0.087*	
H11C	0.6892	−0.1611	0.4903	0.087*	
N1	0.8572 (5)	0.2944 (3)	0.2831 (3)	0.0750 (10)	
Br1	0.79897 (5)	0.04122 (4)	0.05323 (3)	0.07015 (19)	

### Atomic displacement parameters (Å^2^)

**Table:**

	*U*^11^	*U*^22^	*U*^33^	*U*^12^	*U*^13^	*U*^23^
C1	0.0373 (19)	0.053 (2)	0.0457 (18)	−0.0111 (16)	0.0080 (14)	0.0050 (16)
C2	0.057 (2)	0.076 (3)	0.0429 (19)	−0.011 (2)	0.0049 (17)	0.0097 (18)
C3	0.071 (3)	0.101 (4)	0.039 (2)	−0.023 (3)	0.0111 (19)	−0.011 (2)
C4	0.069 (3)	0.069 (3)	0.057 (2)	−0.013 (2)	0.026 (2)	−0.022 (2)
C5	0.053 (2)	0.050 (2)	0.052 (2)	−0.0022 (17)	0.0165 (16)	−0.0044 (16)
C6	0.0321 (17)	0.049 (2)	0.0398 (16)	−0.0060 (14)	0.0106 (14)	−0.0021 (14)
C7	0.0353 (17)	0.043 (2)	0.0452 (17)	0.0012 (15)	0.0058 (14)	−0.0036 (15)
C8	0.0381 (18)	0.043 (2)	0.0412 (17)	0.0057 (16)	0.0041 (14)	0.0000 (15)
C9	0.062 (2)	0.048 (2)	0.0392 (18)	0.005 (2)	0.0082 (16)	0.0103 (16)
C10	0.051 (2)	0.062 (2)	0.0444 (18)	0.0072 (17)	−0.0035 (15)	−0.0007 (15)
C11	0.055 (2)	0.053 (2)	0.065 (2)	−0.0032 (18)	0.0013 (18)	0.0105 (18)
N1	0.099 (3)	0.059 (2)	0.068 (2)	−0.011 (2)	0.0173 (18)	0.0085 (17)
Br1	0.0798 (3)	0.0886 (4)	0.0435 (2)	0.0105 (2)	0.01345 (18)	−0.00341 (19)

### Geometric parameters (Å, º)

**Table:**

C1—C2	1.374 (4)	C7—C8	1.334 (4)
C1—C6	1.409 (4)	C7—H7	0.9300
C1—C11	1.495 (5)	C8—C9	1.422 (5)
C2—C3	1.371 (6)	C8—C10	1.486 (4)
C2—H2	0.9300	C9—N1	1.143 (4)
C3—C4	1.364 (6)	C10—Br1	1.950 (3)
C3—H3	0.9300	C10—H10A	0.9700
C4—C5	1.379 (5)	C10—H10B	0.9700
C4—H4	0.9300	C11—H11A	0.9600
C5—C6	1.378 (4)	C11—H11B	0.9600
C5—H5	0.9300	C11—H11C	0.9600
C6—C7	1.470 (4)		
			
C2—C1—C6	117.9 (3)	C8—C7—H7	116.6
C2—C1—C11	120.2 (3)	C6—C7—H7	116.6
C6—C1—C11	121.8 (3)	C7—C8—C9	121.4 (3)
C3—C2—C1	121.7 (4)	C7—C8—C10	122.1 (3)
C3—C2—H2	119.2	C9—C8—C10	116.4 (3)
C1—C2—H2	119.2	N1—C9—C8	177.2 (4)
C4—C3—C2	120.2 (3)	C8—C10—Br1	111.6 (2)
C4—C3—H3	119.9	C8—C10—H10A	109.3
C2—C3—H3	119.9	Br1—C10—H10A	109.3
C3—C4—C5	119.8 (4)	C8—C10—H10B	109.3
C3—C4—H4	120.1	Br1—C10—H10B	109.3
C5—C4—H4	120.1	H10A—C10—H10B	108.0
C6—C5—C4	120.5 (3)	C1—C11—H11A	109.5
C6—C5—H5	119.8	C1—C11—H11B	109.5
C4—C5—H5	119.8	H11A—C11—H11B	109.5
C5—C6—C1	119.9 (3)	C1—C11—H11C	109.5
C5—C6—C7	121.1 (3)	H11A—C11—H11C	109.5
C1—C6—C7	119.0 (3)	H11B—C11—H11C	109.5
C8—C7—C6	126.7 (3)		
			
C6—C1—C2—C3	1.8 (5)	C2—C1—C6—C7	179.2 (3)
C11—C1—C2—C3	−179.6 (3)	C11—C1—C6—C7	0.6 (4)
C1—C2—C3—C4	−0.4 (5)	C5—C6—C7—C8	41.9 (5)
C2—C3—C4—C5	−0.9 (5)	C1—C6—C7—C8	−139.4 (3)
C3—C4—C5—C6	0.5 (5)	C6—C7—C8—C9	3.9 (5)
C4—C5—C6—C1	1.0 (5)	C6—C7—C8—C10	−177.8 (3)
C4—C5—C6—C7	179.6 (3)	C7—C8—C10—Br1	−114.5 (3)
C2—C1—C6—C5	−2.1 (4)	C9—C8—C10—Br1	63.9 (3)
C11—C1—C6—C5	179.3 (3)		

### Hydrogen-bond geometry (Å, º)

Cg1 is the centroid of the C1–C6 ring

**Table:**

*D*—H···*A*	*D*—H	H···*A*	*D*···*A*	*D*—H···*A*
C7—H7···*Cg*1^i^	0.93	2.97	3.654 (7)	131
C11—H11*B*···*Cg*1^ii^	0.96	2.86	3.699 (1)	146

Symmetry codes: (i) −*x*+1, −*y*, −*z*+1; (ii) −*x*+2, −*y*, −*z*+1.
